# The Best of Two Worlds to Promote Healthy Cognitive Aging: Definition and Classification Approach of Hybrid Physical Training Interventions

**DOI:** 10.2196/56433

**Published:** 2024-07-31

**Authors:** Fabian Herold, Paula Theobald, Thomas Gronwald, Navin Kaushal, Liye Zou, Eling D de Bruin, Louis Bherer, Notger G Müller

**Affiliations:** 1 Research Group Degenerative and Chronic Diseases, Movement Faculty of Health Sciences Brandenburg University of Potsdam Potsdam Germany; 2 Institute of Interdisciplinary Exercise Science and Sports Medicine Hamburg Germany; 3 Department of Health Sciences, School of Health & Human Sciences Indiana University Indianapolis, IN United States; 4 Body-Brain-Mind Laboratory Shenzhen University Shenzhen China; 5 Department of Health Sciences and Technology Institute of Human Movement Sciences and Sport ETH Zürich Zürich Switzerland; 6 Department of Neurobiology, Care Sciences, and Society Karolinska Institute Stockholm Sweden; 7 Department of Health OST - Eastern Swiss University of Applied Sciences St Gallen Switzerland; 8 Montreal Heart Institute Montreal, QC Canada; 9 Department of Medicine Université de Montreal Montreal, QC Canada; 10 Centre de Recherche de l’Institut Universitaire de Geriatrie de Montreal Montreal, QC Canada

**Keywords:** physical activity, dementia prevention, cognitive health, hybrid: aging in place, active, exercises, exercising, healthy lifestyle, dementia, dementia onset, dementia care, preventive, prevention, cognitive health, cognition, cognitive, hybrid, hybrid model

## Abstract

A healthy lifestyle can be an important prerequisite to prevent or at least delay the onset of dementia. However, the large number of physically inactive adults underscores the need for developing and evaluating intervention approaches aimed at improving adherence to a physically active lifestyle. In this regard, hybrid physical training, which usually combines center- and home-based physical exercise sessions and has proven successful in rehabilitative settings, could offer a promising approach to preserving cognitive health in the aging population. Despite its potential, research in this area is limited as hybrid physical training interventions have been underused in promoting healthy cognitive aging. Furthermore, the absence of a universally accepted definition or a classification framework for hybrid physical training interventions poses a challenge to future progress in this direction. To address this gap, this article informs the reader about hybrid physical training by providing a definition and classification approach of different types, discussing their specific advantages and disadvantages, and offering recommendations for future research. Specifically, we focus on applying digital technologies to deliver home-based exercises, as their use holds significant potential for reaching underserved and marginalized groups, such as older adults with mobility impairments living in rural areas.

## Introduction

Dementia is a brain disease that is characterized by an acquired loss of cognitive abilities in different domains which is sufficiently severe to interfere with both activities of daily living and the ability of an individual to live independently [1,2]. As a result, individuals affected by dementia cause high health care expenditures [3-6]. Given the expected worldwide rise in dementia cases from 57.4 million cases in 2019 to 152.8 million cases in 2050 [7], it seems reasonable to assume that the already relatively high health care–related expenditures for dementia (ie, US $1313.4 in 2019 [3]) will dramatically increase in the upcoming years. Thus, preventing dementia should be a priority of public health actions [8,9]. Based on the evidence that the individual dementia risk is substantially influenced by several lifestyle factors (eg, regular engagement in physical activity), a positive modification of these is important to lower the global prevalence and, in turn, the societal and economic costs of dementia [3,8,9]. This assumption is reinforced by the fact that the effectiveness of pharmacological interventions to treat dementia is limited [10,11]. Although lifestyle-related interventions (eg, targeting physical activity, sedentary behavior, diet, or sleep) can be effective and implemented along the entire continuum of dementia [9,12-14], preventive approaches should focus on healthy middle-aged and older adults, and preclinical stages of dementia including adults at-risk (eg, adults with subjective cognitive decline [SCD], mild cognitive impairment [MCI], or with the motoric cognitive risk syndrome [MCR]) [9,15-20] to avoid that cognitive impairment and neurodegenerative changes of the central nervous system become too serious which, in turn, might negatively influence adherence and increase the cost of the implementation of the intervention. At-risk groups for dementia comprise, among others, adults with SCD who have a self-reported or informant-reported worsening of cognitive performance (especially memory) but normal performance on specific clinical cognitive tests [21,22], adults with MCI who have objective cognitive deficits determined by a clinical cognitive test battery [23-25], and adults with MCR who have subjective cognitive complaints and a slow gait speed [17,20,26-28]. SCD, MCI, and MCR are preclinical stages of dementia that are unrelated to an acute event, do not interfere with activities of daily living, and thus allow the individual to live independently [17,20,21,24-27]. Current estimates suggest that worldwide 315 million individuals aged 50 years or older are in a preclinical stage of dementia [29] emphasizing the pressing need to develop and implement appropriate intervention strategies to prevent or at least delay the onset of dementia in the general and at-risk populations.

In the literature, there is some evidence showing that among other factors such as sedentary behavior, sleep, and diet, especially regular physical activity including planned and structured forms such as physical exercise and physical training [6,30-35] can be an important factor to preserve cognitive functions [36-44] and to prevent or at least delay the onset of dementia in older adults [6,9,45,46] although future high-quality randomized controlled trials (RCTs) are required to further substantiate the evidence in this direction [47,48]. Hence, the high prevalence of physical inactivity among the general adult population [6,49] which increases with age [50-52] and low cognitive status [53], necessitates the development of new, more efficient, and sustainable intervention approaches to preserve and maintain cognitive health across the life span, especially in adults-at-risk to develop dementia (eg, adults with MCI) [9,16].

Concerning new approaches to support healthy cognitive aging, there is the opinion in the literature that using digital technologies is a promising and cost-saving option that can support healthy aging in place (eg, by providing lifestyle interventions) [54-56]. In this context, digital technologies can be a valuable instrument to remotely assess and monitor changes in cognitive health [57-63] and to facilitate positive lifestyle changes [57,63-65] by fostering a higher level of regular physical activity (eg, via wearable physical activity trackers [66,67] or via apps or online classes to remotely deliver home-based exercise sessions [64]), which is especially relevant for adults living in rural and remote areas [64,68-71].

Digital technologies can be applied in a wide range of contexts and in recent years they have become a popular instrument for delivering physical exercises and physical training to the general population [64,72-75]. For instance, in center-based exercise sessions, the application of digital technologies encompasses but, is not limited to (1) monitoring and controlling exercise intensity (eg, via wearables recording heart rate) [76-78], or (2) delivering specific physical exercises (eg, via exergames [79-81] or videoconference-based online classes, in which the exercise professional is remotely connected to the groups of trainees being located in a center [82]). However, digital technologies provide also several advantages making them extremely well-situated for delivering home-based exercise sessions. This assumption is supported by the latest editions of the Worldwide Survey of Fitness Trends revealing that digital applications such as wearable technologies, online training, and mobile exercise apps are among the top 20 fitness trends in recent years [72-75]. More specifically, the Worldwide Survey of Fitness Trends also showed that exercising at home is the number 2 trend for 2022 [73] probably due to the consequences of the COVID-19 pandemic (eg, home confinement). Even from a more general point of view, exercising at home provides some benefits for older adults (eg, no need to commute to a training facility) because home-based approaches can help to circumvent frequently mentioned barriers to engaging in planned and structured forms of physical activity (eg, the lack of opportunity or transport [83-86]).

Compared with traditional options, the use of digital technologies can be a promising alternative (1) to remotely recruit and assess a large number of (diverse) participants (eg, in decentralized trials instead of centralized trials) [87], and (2) to deliver home-based interventions (eg, via video capsules instead of booklets) [64]. In particular, remotely delivered home-based interventions via digital technologies can provide several advantages such as better visualization of exercises or gamification (eg, exergames) as compared with nondigital solutions (eg, booklets) [64]. Thus, digital technologies can play an important role in reducing inequalities arising from various reasons (eg, living in a rural area without a driving license) which, in turn, might contribute to increased robustness of the findings of the trials because of the inclusion of larger and more diverse samples of older adults at-risk or with cognitive impairment [87-91]. Further empirical data supporting our claims have been published recently [90-93]. However, using digital technologies and home-based interventions does not come without limitations [87,94]. For instance, home-based physical exercise sessions provided via digital technologies are typically associated with lower levels of supervision, and social interaction (ie, social support and relatedness such as the feelings of belonging to a group) [95] although the level of supervision and social interaction depends on the type of digital technology used for that purpose [64]. Given that the social factors are important for adhering to an intervention [96], the unique characteristics of different types of digital technologies being used for delivering home-based physical exercises should be considered when designing and implementing future trials [87] and suggest that establishing a classification framework to improve the structuring of knowledge is required (eg, as started in [64]) to better inform the application in practical settings.

Since factors such as supervision, social support, and relatedness can be more easily promoted in center-based physical exercise sessions (eg, in clinical settings) because of the interaction with health professionals (eg, certified trainers, physiotherapists) and peers, several studies have combined both center-based physical exercise sessions and home-based physical exercise sessions. For instance, especially in rehabilitative settings such as cardiac rehabilitation [97-99] or orthopedic rehabilitation (eg, hip or knee osteoarthritis [100]) interventions that combine center- and home-based physical exercise sessions are widely used. Such interventions are typically referred to as hybrid. With regard to healthy cognitive aging, hybrid physical training interventions have been somewhat neglected [64] but have been highlighted as a promising option for future studies (eg, for older adults at risk or with cognitive impairment) [87]. Moreover, neither a generally accepted definition nor a classification framework for hybrid physical training interventions is available. As such a classification framework allows for better structuring of knowledge and might foster research in the field of healthy cognitive aging (eg, by increasing the awareness of research in which different types of hybrid physical training interventions exist), this article aims to inform the reader about this intervention approach by providing a definition and classification approach of hybrid physical training and by narratively summarizing the current state of the literature. In accordance with the literature [31-35,101], we use in the forthcoming sections of this article the term “physical training” to refer to chronic forms of planned and structured forms of physical activity. Furthermore, we focus on hybrid physical training approaches that use digital technologies for delivering home-based exercises because their application holds great potential to reach needy but underserved and marginalized groups (eg, older adults with mobility impairments living in rural areas). The latter is related to the facts (1) that reaching and including such cohorts is highly relevant from a public health perspective given the paucity of research in this direction and the existing rural health disparities [68-71,102], and (2) that research in this direction will improve our understanding of whether the effects of physical training on cognitive health typically observed in laboratory setting extend to ecologically valid settings (eg, community-dwelling older adults living in rural areas) for which the evidence base is currently relatively scant [103].

## Hybrid Physical Training: A Definition and Classification Approach

### Overview

Hybrid interventions have been recently highlighted as a promising option to improve health-related outcomes, especially in rehabilitation settings [87,98,100,104] but, to the best of our knowledge, neither a generally accepted definition nor a classification approach exists. To address this issue and avoid ambiguity which can be a major source of difficulty in scientific communication, we define the term “hybrid physical training” as an intervention that combines center-based physical exercise sessions, that is, conducted in a clinical setting (eg, health care facility) or community setting (eg, local gym) [105] and home-based physical exercise sessions, that is, undertaken inside or within the immediate vicinity of the home (eg, apartment, park) [100,104,105].

To derive a classification approach for different types of hybrid physical training interventions, we orient on recommendations on constructing, conducting, and analyzing physical interventions [105,106] and recent literature in this field [64,107]. In this context, we propose that hybrid physical training interventions can be differentiated based on (1) temporal characteristics, that is, how the center-based exercise sessions and home-based physical exercise sessions are combined, and (2) specific intervention-related characteristics, that is, how center-based physical exercise sessions and home-based physical exercise sessions are delivered.

### Temporal Characteristics

**Figure 1:**
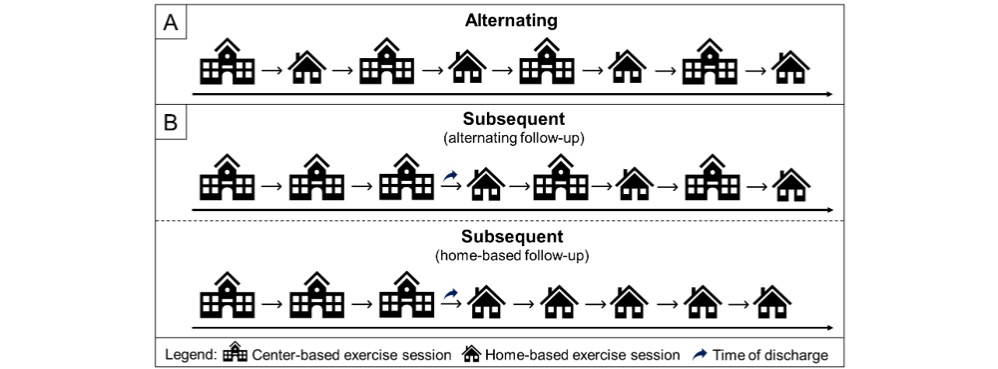
Overview of possible temporal organizations of hybrid physical training interventions which can be divided into (A) alternating hybrid physical training interventions and (B) subsequent hybrid physical training interventions.

**Figure 2:**
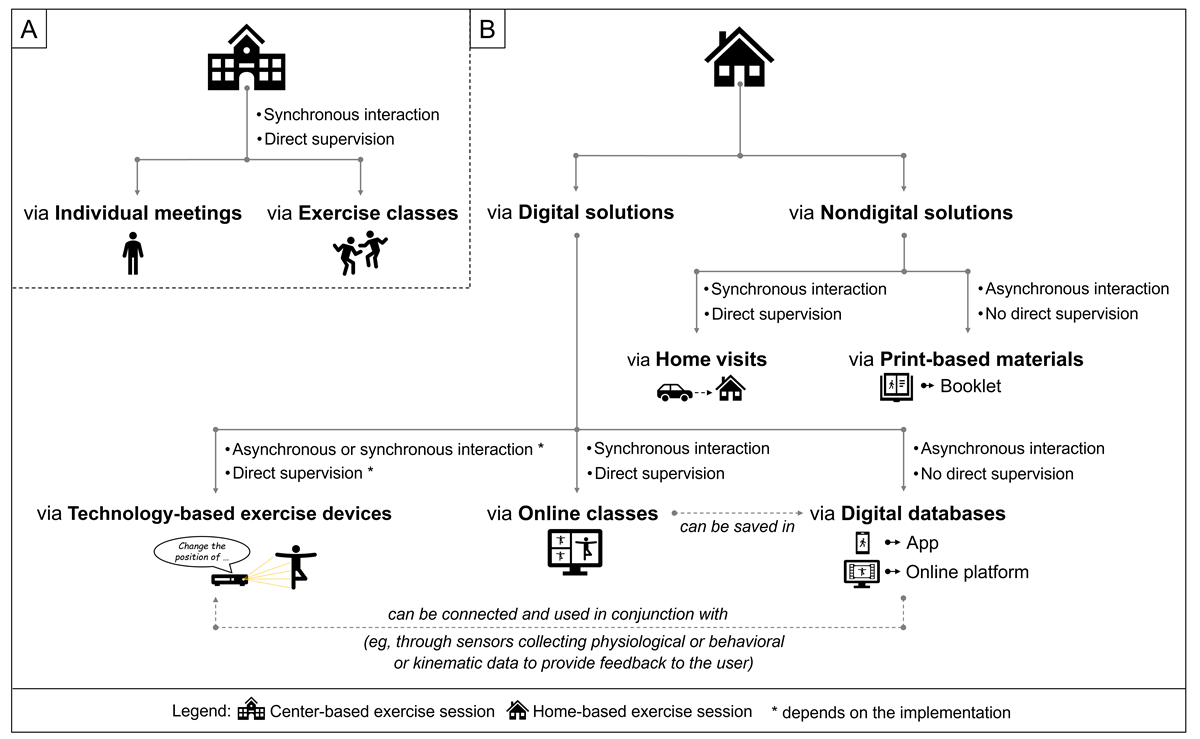
Overview by which means (A) center-based physical exercise sessions and (B) home-based physical exercise sessions can be delivered.

Concerning the temporal characteristics, we propose that hybrid physical training can be differentiated as (1) alternating hybrid physical training interventions, and (2) subsequent hybrid physical training interventions (Figures 1A and 1B). First, in alternating hybrid physical training interventions, center-based physical exercise sessions are alternated with home-based physical exercise sessions. While this alternation provides, on the one hand, the advantage that the participant has frequent contact with health professionals and peers fostering higher levels of supervision and social interaction (ie, especially in center-based physical exercise sessions) that might positively influence compliance and adherence to the intervention [108], it is, on the other hand, a clear disadvantage because it demands the participants to commute to attend the center-based physical exercise sessions. However, the alternating hybrid physical training can also unburden the participants because it reduces the number of travels (eg, 1 travel per week if there are 2 sessions per week) and thus allows for a higher frequency (eg, 2 exercise sessions instead of 1 per week), which has been linked to larger improvements of cognitive performance in adults aged 50 years or older [44]. Second, in subsequent hybrid physical training interventions, a phase of center-based exercise sessions is followed by a phase of home-based exercise sessions (Figure 1B). This type of intervention can be further divided into (1) subsequent hybrid physical training interventions with alternating follow-up or (2) subsequent hybrid physical training interventions with home-based follow-up. The advantages and disadvantages of subsequent hybrid physical training interventions strongly depend on the type of follow-up (Figure 1B) and the implementation of the home-based physical exercise sessions (Figure 2B). For instance, subsequent hybrid training interventions with alternating follow-up can enable relatively high levels of supervision and social interactions due to frequent contact with health professionals and peers during the center-based physical exercise sessions. On the downside, to attend center-based physical exercise sessions, the participants are obliged to commute to the training facility, which is in some studies mentioned as a barrier, especially by older women [84]. A home-based follow-up offers the advantage that the participants are not obliged to commute to the training facility but comes at the cost of lower levels of supervision (eg, by the health professional) and limited social interactions (ie, with peers) during the home-based physical exercise sessions in the follow-up, although the latter can be influenced by the implementation (eg, higher levels of supervision and social interaction may be achievable via online classes [64]; Figure 2B).

### Specific Intervention-Related Characteristics

Based on a recent systematic review of our group, in which we propose a classification approach for digital- and home-based physical training interventions [64], we used the type of interaction and the level of supervision to differentiate between different types of hybrid physical training interventions.

Before we discuss the different types of hybrid physical training interventions and their strengths and limitations in more detail, we provide the reader with a brief description of the different types of interaction and the different levels of supervision.

Regarding the type of interaction, one can distinguish between asynchronous interaction and synchronous interaction. Synchronous interaction refers to an interaction that occurs in real-time, whereas asynchronous interaction refers to a decoupled interaction (eg, at different times) [64,109-113]. Thus, a synchronous interaction allows for the immediate exchange of information (eg, immediate feedback on specific exercises—direct supervision) but is limited concerning time flexibility [64,109]. Vice versa, asynchronous interaction provides the advantage of on-demand information exchange at the convenience of the participating parties but bears—as a consequence of the decoupled communication—the risk that specific information is not received on time (eg, short-term adjustment of a physical exercise session due to quality of exercise execution or acute pain of the trainee is difficult) [64,109].

Regarding supervision, one can distinguish between direct supervision (ie, each exercise session is monitored by a health professional or digitally via sensors allowing for immediate feedback on exercise execution), general supervision (ie, no direct supervision of each exercise session but regular in-person or web-based contact with a health professional to support the trainee; also referred to as “facilitated”), and no supervision (ie, the trainee has no direct contact with a health professional throughout the intervention period; also referred to as “unsupervised”) [64,105]. In general, there is some evidence suggesting that higher levels of supervision (ie, direct supervision) can facilitate adherence to physical training interventions [96,114,115] although, on the downside, higher levels of direct supervision can increase the cost of the intervention (ie, to pay the health professional who supervises the physical exercise sessions) and lower the time flexibility (ie, as result of the synchronous interaction) [96].

As shown in Figure 2A, center-based exercise sessions are typically supervised and are based on synchronous interactions with health professionals and other peers. In addition, center-based exercise sessions can be conducted individually or in a group. Individual meetings provide the advantage that a high level of individualization can be achieved because of close contact with the health professional. In individual meetings, the social interaction with peers, in contrast, to exercise classes, might be somewhat limited. In this context, it is worth noting that in older adults’ social factors such as loneliness are associated with worse cognitive functioning [116-119] and increased risk of dementia [8,120-123]. Furthermore, in a cross-sectional study of older adults with MCI, a higher level of perceived loneliness was observed as compared with age-, sex-, and education-matched controls with slightly less pronounced cognitive deficits [124]. In addition, social factors are also important promotors for regular engagement in specific forms of physical training (eg, sports) [84,125-127] and long-term adherence to interventions [96,128]. Accordingly, promoting social participation (eg, via group-based physical training), especially in at-risk cohorts for dementia (eg, older adults with MCI), seems to be an important factor for fostering adherence to physical training interventions [96,128], preventing cognitive decline, and potentially reducing the risk of dementia [129]. Indeed, there is evidence that in healthy older adults’ group-based physical training interventions can counteract social isolation and loneliness [130,131]. Moreover, there is evidence from cross-sectional studies that older adults who exercise alone and exercise with others (eg, team or partner sports activities) showed better cognitive performance and have a lower risk of cognitive impairment, but that exercising with others is slightly more beneficial than exercising alone [132-134] even after adjusting for important influencing factors (eg, accelerometer-derived physical activity or time spent for other social engagements [133]). Based on these findings, group-based physical training, which can lead to a higher level of social interaction (ie, with peers) and perhaps trigger specific psychosocial-related changes [133] on multiple levels of analysis [129,135-137], might provide some additional benefits to preserve cognitive health as compared with exercising alone or without peers (eg, individual meetings).

The level of social interaction (eg, with peers) depends, among other factors, on the type of digital technology that is used to deliver the home-based physical exercise sessions. As shown in Figure 2B, home-based physical exercise sessions can be broadly categorized based on the instruments being used to deliver and prescribe the physical exercises—namely digital and nondigital solutions.

## Nondigital Solutions to Deliver Home-Based Physical Exercise Sessions

We propose that nondigital solutions can be divided into two different types: (1) the term “home visits” refers to physical training interventions in which the health professional provides the physical training at the participant’s home. Although home visits ensure a high level of individualization because of the direct supervision (ie, synchronous interaction), they are relatively costly as the health professional provides 1:1 supervision and needs to commute to the participant’s home [96]; and (2) the term “print-based materials” characterize interventions in which the exercise and training prescription is provided via nondigital solutions such as booklets. This type of intervention provides the advantage that it can be disseminated on a broad scale even in low-resource settings (eg, with limited access to digital technologies). Owing to the asynchronous interaction, this intervention type is, in general, unsupervised, and thus it cannot be controlled for a proper exercise execution [64].

## Digital Solutions to Deliver Home-Based Physical Exercise Sessions

In general, digital solutions, as compared with nondigital solutions, can be less costly (ie, online communication in comparison to home visits), allow for better visualization of how the exercises should be conducted (ie, by video instructions which provide more appropriate exercise execution cues than print-based material), allow clinician-based digital care to replace or complement traditional in-person visits in a health care center due to synchronous video or audio visits, and potentially allow for a “gamification” of exercise programs (ie, in comparison to print-based materials) [64]. For instance, there is some evidence that “gamification” can positively influence the promotion of physical activity [138,139]. Because of the aforementioned advantages especially digital solutions to deliver home-based exercise sessions within a hybrid physical training intervention seem to be a promising approach to facilitate the implementation and improve the effectiveness of such an intervention program. In the following, we will discuss in more detail the different types of digital solutions to deliver home-based physical training using a classification approach that has been previously proposed by our group [64]. For a more comprehensive overview of the physical exercise and training characteristics and effectiveness of digital and home-based physical training interventions to improve cognitive functions, we refer the interested reader to a recent systematic review of our group [64].

First, the term “digital databases” comprises digital and home-based physical training interventions that rely on the storage of the exercise prescriptions (eg, via video capsules) on specific digital media platforms (eg, smartphone or tablet-based apps) [64] and thus, can be considered as the digital alternative to print-based materials (eg, booklets). This type of digital and home-based physical training provides the advantage that it can be delivered to a relatively large number of individuals and allows for on-demand training based on asynchronous interaction [64]. A drawback of asynchronous interaction, the inherent feature of digital databases, typically no direct supervision of the single exercise sessions during the home-based training exists [64]. Concerning the implementation of digital databases to support healthy cognitive aging, different digital platforms or applications such as YouTube video capsules [140,141], DVDs [142,143], and tablet-based [144-147] or smartphone-based app [148] have so far been used to deliver a wide range of different types of physical exercises (eg, endurance, resistance, flexibility, or coordinative exercise such balance, or dancing exercises) to diverse cohorts including healthy-middle-aged and older adults [141] or middle-aged and older adults with MCI [144], cardiovascular disorders [140], or Parkinson disease [147,148]. Given that hand-eye coordination, visual acuity, and mental acuity are frequently reported barriers to the use of digital health technologies in older adults [149], commercially available devices with voice-controlled intelligent personal assistants (eg, Amazon Alexa) can be a promising alternative to remotely delivering physical exercises because the voice control helps older adults to access the exercise instructions more easily. Indeed, several small-scale studies (n=15) showed that applying voice-controlled digital solutions to remotely deliver physical exercise instruction and feedback is feasible in older adults [150-152] and is associated with high completion adherence (ie, 100%) and attendance adherence rates (ie, 115% of the prescribed exercise sessions) [152]. Thus, investigating the cognitive health effects of remotely delivered physical training, which uses devices with voice-controlled intelligent personal assistants (eg, Amazon Alexa), seems to be a promising area for further investigation.

Second, the term “online classes” refers to digital and home-based physical training interventions that are characterized by a synchronous and remote interaction of the trainees with the health professional via videoconference software [64]. Hence, it can be considered as some kind of digital alternative to home visits, typically used to supervise a single trainee or a group of trainees who exercise individually at their homes. In this context, it is worth noting that online classes can be delivered via static videoconferencing (eg, PC with camera) or dynamic videoconference (eg, via a mobile telepresence robot [MTR]) [153,154]. MTRs are typically wheeled devices that are remotely controlled by the operator (eg, exercise professional) and which allows them to be present through the robotic embodiment at remote locations, move throughout the environment, and interact with persons (eg, trainee) in the environment by 2-way audio-visual communication [154,155]. Using MTRs to deliver online classes provides the advantage of superior mobility that enables the remote controller (ie, exercise professional) to change the point of view (eg, when the trainee leaves the field of view of the camera) and thus allows for better direct supervision of single trainees or the direct supervision of several trainees exercising as a group at the same location (eg, gym in a retirement home) as compared with static videoconferencing [153,154]. While online classes provide a relatively high level of direct supervision, they are, on the downside, limited with regard to the number of trainees (eg, to ensure an appropriate level of direct supervision) and time flexibility (ie, because of fixed time schedules) [64]. In intervention studies that implement physical training via online classes (ie, static videoconference) to support healthy cognitive aging, different types of directly supervised physical exercises including endurance exercises such as cycling [156], resistance exercises [157], chair-based motor-cognitive exercises [158], or Tai Ji Quan [159] were remotely delivered to healthy older adults [157] or older adults with SCD [156], MCI [159], and Alzheimer disease [158]. In future studies, dynamic videoconference solutions using MTR could be a promising alternative to static videoconference because of their specific advantages over static videoconferencing (eg, the opportunity to change the point of view by moving the robot), and the findings (1) that older adults found MTRs useful, easy to use, and pleasant for remote supervision of physical exercises [153] and (2) that MTRs can be as effective as face-to-face interaction with an exercise professional to learn a new motor cognitive task within a single session [154]. Although these findings are encouraging, future long-term studies are needed to substantiate the evidence on the applicability of MTRs to deliver and supervise physical exercise sessions before they can be unreservedly recommended.

Third, the term “technology-based exercise devices” covers a wide range of instruments that can be used to remind individuals to be physically active (eg, just-in-time adaptive physical activity interventions using real-life data of wearables such as smartwatches or smartphones to provide real-time recommendations for opportunities to be physically active [160-162]) or that are used to conduct physical exercises (eg, virtual reality–enhanced stationary ergometer [163-166]) [64]. Such technology-based exercise devices include but are not limited to exergames. The term “exergames” (ie, also referred to as active video games or gamercizing) is a portmanteau consisting of the terms “physical exercise” and “gaming” [167] and refers to technology-driven physical activities in a gaming environment requiring participants to become physically active to play the game [168-170]. Technology-based exercise devices offer the advantage that they allow for on-demand training, but the level of direct supervision is constrained by the implementation of technical features (eg, integration of different sensors or camera-based information allowing for the assessment of behavioral, physiological, and kinematic data) [64]. In this regard, technology-based exercise devices fulfill the criteria of asynchronous interaction (eg, can be used on-demand) and synchronous interaction (ie, can provide immediate feedback to the user through integrated sensors or camera-based information), although the latter strongly depends on the technological features of the device [64]. Studies aiming to support healthy cognitive aging via technology-based exercise devices typically used specifically designed step-based exergames [171-178] or cycling-based exergames [163-166] in older adults with different health statuses (eg, in healthy older adults [171-175,178], older adults with Parkinson disease [163,164,176], multiple sclerosis [177], or MCI [165,166]), whereas the application of other technology-based exercise devices (eg, wearables such as activity trackers) is less common [64]. Given (1) that wearables (eg, activity trackers) can increase the level of regular physical activity [66,67] and (2) that physical activity is an important factor for preserving cognitive performance [36-44] and lowering dementia risk in older adults [6,9,45,46], wearables (eg, to deliver just-in-time adaptive physical activity interventions [160-162]) may hold some promise to support healthy cognitive aging, although they have so far been underused to prescribe and monitor physical activity in the context of healthy cognitive aging [64].

In summary, we proposed a classification framework and provided definitions for different types of nondigital and digital solutions to remotely deliver home-based physical exercise sessions that can guide future research in the field of healthy cognitive aging. In this context, we wish to acknowledge that in practical and research settings different nondigital and digital solutions (eg, home visits and online classes), different nondigital solutions (eg, home visits and booklet [179]), or different digital solutions (eg, digital database and online classes [180,181]) can be combined to deliver home-based physical exercises but we intentionally refrained from discussing or visualizing all possible combinations (Figure 2) for reasons of clarity and comprehensibility.

## Challenges of Implementing Hybrid Physical Training Interventions Using Digital Technologies to Deliver Physical Exercise Sessions Remotely

Digital technologies provide several advantages when they are used to remotely deliver physical exercise sessions (see *Digital Solutions to Deliver Home-Based Physical Exercise Sessions* section) [64]. However, the use of digital technologies in older adults without and with cognitive impairment is also associated with some challenges including but not limited to (1) infrastructural- and device-related factors (eg, absence of stable internet coverage and unequal availability of digital technology or devices in rural or socioeconomically deprived areas), and (2) user-related factors (eg, digital literacy, privacy concerns, perceived usefulness and ease of use of the digital application, support of the social network) [57,94,150,182-188]. In this context, there is, on the one hand, some evidence that older adults with cognitive complaints or decline and who did not regularly use a computer show a lower engagement in a digital intervention aiming to reduce dementia risk through lifestyle changes [189]. On the other hand, several studies provided evidence that digital technologies can be successfully used to remotely deliver physical exercise sessions in diverse cohorts [64] including healthy older adults [142,143,145,171-175,190-192], older adults with MCI [144,159,180] and SCD [146], and even older adults with dementia [193,194]. Although the latter evidence clearly supports the feasibility of such approaches, future studies aiming to implement hybrid physical training interventions by using digital technologies to remotely deliver home-based exercise sessions should consider (1) infrastructural prerequisites (eg, in the absence of a stable internet coverage the application of digital databases instead of online classes to remotely deliver physical exercise sessions) and (2) use a user-centered and participatory approach when designing and tailoring the intervention to the specific needs of older users (eg, group-based physical training to ensure higher levels of social interaction) [57,65,70,91,92,195]. In addition, from a theoretical point of view and compared with a fully remote implementation, hybrid physical training interventions (ie, with alternating center- and home-based physical exercise sessions) provide the advantage that the regular social interaction between health professional, trainee, and peers during the center-based physical exercise sessions might lead to the development of a social network that allows to more easily overcome barriers or difficulties emerging from the use of digital technologies to remotely deliver the home-based physical exercise sessions. For instance, the possibility of receiving social and technological support, if needed, by the health professional and peers during center visits might foster the implementation and adherence to hybrid physical training interventions as compared with fully remote interventions which are characterized by somewhat looser social interactions. This assumption is supported by the fact that the lack of support is often named as a barrier to the use of digital technologies by older adults without and with cognitive impairment [182,185], although future empirical studies are needed to verify (or refute) our idea that such a lack of technological support can be reduced by hybrid physical training interventions.

Another important point that needs to be considered when providing home-based physical training with and without digital solutions is safety. In general, there is evidence that home-based physical training interventions are safe [196] even when using digital solutions [64] or when conducted in vulnerable cohorts such as adults with cardiac diseases [197,198]. In this context, the findings of a recent systematic review and meta-analysis provide evidence that there is no difference in safety-related measures (eg, dropouts) between home- and center-based physical training [196] buttressing the assumption that home-based physical training is a safe alternative to center-based physical training. However, intuitively we suggest that a higher level of direct supervision (eg, online classes) should be used to further minimize the risk of negative health events in vulnerable groups (eg, older adults with chronic diseases and high risk of falls). However, future research studying the influence of different moderators on safety outcomes (eg, level of supervision, specific setting, and trainees’ characteristics such as age and health status) is needed to provide evidence-based and more nuanced recommendations in terms of the safety of remotely delivered and home-based physical training.

## Current State of Evidence on Hybrid Physical Training Interventions to Support Healthy Cognitive Aging

### Overview

Hybrid physical training is a novel and promising form of delivering physical training to support healthy cognitive aging and thus the evidence of its effectiveness is, as far as we know, relatively scant [64] because only a small quantity of trials has been conducted to study the effects of hybrid physical training approaches on cognitive performance [190,199,200]. The characteristics and findings of these studies will be briefly summarized in the following.

### Alternating Hybrid Physical Training

In the RCT of Auerswald et al [190] the effect of a 10-week hybrid physical training in alternating mode (Figure 1A) on different health-related outcomes (ie, including cognition) was investigated in a larger sample of healthy older adults (n=551, included in the final statistical analysis). The participants were randomly allocated to a waitlist control group (CG), an intervention group 1 (IG1) that received physical exercise prescription via print-based material or digital database, or an intervention group 2 (IG2) that received physical exercise prescription via digital database and also used a wearable (ie, physical activity tracker) [190]. The physical exercise prescription for the home-based program was individualized based on the individual fitness level of the participants and follows the recommendations of the World Health Organization for regular physical activity in adults [6,201,202] (eg, balance and flexibility exercise 2 times per week, resistance exercises at 2 nonconsecutive days per week, and endurance exercise for at least 150 minutes with moderate intensity) [190]. In IG1 and IG2, the home-based physical exercises were complemented by once-weekly center-based group meetings in which health education and group-based physical exercise sessions were performed [190]. In IG2, the wearable was used to provide the participants with objective feedback on the regular physical activity level [190]. Although the participants in the IG2 and CG showed better executive functions (ie, faster reaction time in the Simon task) in the posttest compared with the pretest, no significant group differences regarding cognitive performance were observed between CG, IG1, and IG2 [190].

Moon et al [200] conducted a 24-week-long RCT in older adults with one or more modifiable dementia risk factors, but no manifest dementia [200]. The participants (n=140, included in the final statistical analysis) were randomized to a CG (ie, receiving general health education), or 1 of 2 multimodal IGs (eg, including elements such as monitoring and management of metabolic and vascular risk factors, cognitive training and social activity, physical exercise, nutritional guidance, and motivational enhancement) that received 3 times a week a 60-minute session of only supervised center-based physical training or alternating hybrid physical training (Figure 1A). The unsupervised home-based physical exercise sessions consisted of endurance, resistance, flexibility, balance, and finger-and-toe exercises that were delivered via booklets or videos (ie, digital database) [200]. In the first 2 months, the hybrid physical training group performed 1 supervised group- and center-based physical exercise session per week (ie, 2 physical exercise sessions per week at home); whereas in the remaining 4 months, 1 supervised group- and center-based physical exercise session was conducted every 2 weeks (ie, 2 home-based physical exercise sessions in a week with a center visit and 3 home-based physical exercise sessions in a week without a center visit) [200]. Regarding cognitive outcomes, the hybrid physical training group showed (1) a better performance in visuoconstruction, attention, and total scale index score in the Repeatable Battery for the Assessment of Neuropsychological Status, and (2) a better score in the Prospective Retrospective Memory Questionnaire (ie, both as compared with the CG) [200].

### Subsequent Hybrid Physical Training With Home-Based Follow-Up

In the randomized controlled pilot study of Wiebking et al [199], which can be classified as subsequent hybrid physical training with home-based follow-up (Figure 1B), a 12-week long intervention (ie, 3 weeks center-based and 9 weeks home-based; 3 exercise sessions per week each lasting 30-40 minutes) was conducted in a small sample (n=21, included in the final statistical analysis) of middle-aged adults with chronic unspecific low-back pain. The participants were randomly allocated to the CG receiving usual care, a unimodal intervention group receiving sensorimotor training (SMT) consisting of stability and resistance exercises, and a multidisciplinary intervention group receiving sensorimotor training supplemented by behavioral therapy (SMT+BT) that includes cognitive distraction during sensorimotor exercises, psychoeducation, and a body scan [199]. The center-based physical exercise sessions were group-based and directly supervised by an exercise professional, whereas the home-based physical exercise sessions were delivered via a digital database (ie, DVD) [199]. Concerning cognitive measures, a faster processing speed (ie, assessed via Trail Making Test A) was observed after the intervention and at the 3-month follow-up only in the SMT+BT group but not in the SMT or the CG group [199].

## Interim Summary

Collectively, the findings of the aforementioned studies suggest that hybrid physical training is a promising approach to fostering healthy cognitive aging. However, the limited number of trials that have studied the effect of hybrid physical training on cognitive performance in the aging population does not allow for drawing more nuanced conclusions (eg, which type of hybrid physical training is most suitable for a specific context and setting). Thus, future research is needed to derive more robust and nuanced conclusions on the effectiveness of specific types of hybrid physical training to support healthy cognitive aging. In this context, our classification framework of hybrid physical training interventions provides researchers with an orientation that can help them to (1) structure findings and knowledge and (2) elucidate which type of hybrid physical training is most suitable for the specific context and setting. In prompting the direction of future research, we provide in the next section recommendations for research priorities concerning hybrid physical training and healthy cognitive aging.

## Recommendations for Future Research

Considering the facts that (1) hybrid physical training interventions, especially those that use digital solutions to provide home-based physical exercise sessions, have been underused to promote planned and structured forms of physical activity in healthy middle-aged and older adults and adults at higher risk to develop dementia (eg, adults with MCI) [64] and (2) hybrid physical training provides some specific advantages depending on their implementation (see Interim Summary section for a detailed discussion), we recommend that future research should aim to answer the following research questions:

- Do the positive effects of physical training on cognitive health and dementia risk, as seen for traditional physical training intervention approaches, extend to hybrid physical training interventions?
- Is the potential effectiveness of hybrid physical training interventions influenced by their implementation (eg, temporal and intervention-related characteristics)?
- Do hybrid physical training interventions, as compared with traditional physical training approaches, improve measures of adherence?
- Are hybrid physical training interventions using digital technology feasible, safe, and effective in special populations with lower digital health literacy (eg, older adults with MCI)?

In the context of the aforementioned research questions, we propose that future studies should especially focus on, but are not limited to, measures of cognitive performance (eg, global cognition and memory), dementia-related risk factors and biomarkers (eg, blood-based parameters), ability to live independently (eg, activities of daily living, life-space mobility), adherence (eg, attendance and completion adherence [64,203,204]), social interaction (eg, social isolation, social connectedness), and cost use. To get a more comprehensive understanding of the neurobiological mechanisms that drive the positive effects of physical activity on cognitive health and to gain a deeper understanding of how physical training interventions can be effectively tailored to fit the individual needs of healthy middle-aged and older adults, and older adults with existing health conditions (eg, MCI), future studies should (1) use a rigorous study design [205-207] including a transparent reporting of the intervention-related characteristics [34,35,106,208], (2) conduct analysis on multiple levels (ie, level 1: changes on a molecular and cellular level, level 2: functional and structural brain changes, and level 3: socioemotional changes) [36,38,64], and (3) consider potential moderators such as sex or genetic status [209-213].

## Conclusions

Hybrid physical training interventions, which consist of both center-based physical exercise sessions and home-based physical exercise sessions, can be a promising option to better tailor physical training-based approaches aiming to promote healthy cognitive aging to the individual needs of a person because they combine the strengths of a center-based approach (eg, supervision and social contact) and a home-based approach (eg, on-demand training), which, in turn, may increase the efficacy of such a form of intervention. To advance research in this direction, this article (1) provides a definition of hybrid physical training, (2) proposes a classification approach of different types of hybrid physical training interventions, (3) discusses the specific advantages and disadvantages of the different types of hybrid physical training interventions, and (4) provides recommendations for future research on hybrid physical training interventions in the context of healthy cognitive aging.

## Abbreviations

CG: control group
IG1: intervention group 1
IG2: intervention group 2
MCI: mild cognitive impairment
MCR: motoric cognitive risk syndrome
MTR: mobile telepresence robot
RCT: randomized controlled trial
SCD: subjective cognitive decline
SMT: sensorimotor training
SMT+BT: sensorimotor training supplemented by behavioral therapy
